# Critical role of β1 integrin in postnatal beta-cell function and expansion

**DOI:** 10.18632/oncotarget.17969

**Published:** 2017-05-18

**Authors:** Jason Peart, Jinming Li, Hojun Lee, Matthew Riopel, Zhi-Chao Feng, Rennian Wang

**Affiliations:** ^1^ Children's Health Research Institute, London, Ontario, N6C 2V5, Canada; ^2^ Department of Pathology, University of Western Ontario, London, Ontario, N6A 3K7, Canada; ^3^ Department of Physiology and Pharmacology, University of Western Ontario, London, Ontario, N6A 3K7, Canada

**Keywords:** β1 integrin, mouse insulin promoter (MIP), glucose tolerance test, Cre recombinase, beta-cell mass

## Abstract

β1 integrin is essential for pancreatic beta-cell development and maintenance in rodents and humans. However, the effects of a temporal beta-cell specific *β1 integrin* knockout on adult islet function are unknown. We utilized a *mouse insulin 1* promoter driven tamoxifen-inducible Cre-recombinase *β1 integrin* knockout mouse model (MIPβ1KO) to investigate β1 integrin function in adult pancreatic beta-cells. Adult male MIPβ1KO mice were significantly glucose intolerant due to impaired glucose-stimulated insulin secretion *in vivo* and *ex vivo* at 8 weeks post-tamoxifen. The expression of *Insulin* and *Pancreatic and duodenal homeobox-1* mRNA was significantly reduced in MIPβ1KO islets, along with reductions in insulin exocytotic proteins. Morphological analyses demonstrated that beta-cell mass, islet density, and the number of large-sized islets was significantly reduced in male MIPβ1KO mice. Significant reductions in the phosphorylation of signaling molecules focal adhesion kinase, extracellular signal-regulated kinases 1 and 2, and v-Akt murine thymoma viral oncogene were observed in male MIPβ1KO islets when compared to controls. MIPβ1KO islets displayed a significant increase in protein levels of the apoptotic marker cleaved-Poly (ADP-ribose) polymerase and a reduction of the cell cycle marker cyclin D1. Female MIPβ1KO mice did not develop glucose intolerance or reduced beta-cell mass until 16 weeks post-tamoxifen. Glucose intolerance remained in both genders of aged MIPβ1KO mice. This data demonstrates that β1 integrin is required for the maintenance of glucose homeostasis through postnatal beta-cell function and expansion.

## INTRODUCTION

Integrins are important regulators of cellular differentiation, migration, maturation, and survival [1–3]. β1 integrin is the major β integrin subunit in pancreatic beta-cells and forms heterodimers with 12 α integrin subunits, which interact with components of the extracellular matrix (ECM) to control intracellular signaling via the focal adhesion kinase (FAK) and extracellular signal related kinase 1 and 2 (ERK1/2) pathways [4]. The role of β1 integrin in maintaining optimal islet function has previously been identified *in vitro* using cell lines and isolated islets. Cultured rat [5–8] and human [4, 9–11] islets have shown that inhibition of β1 integrin reduced insulin secretion [8, 10] and islet adhesion to ECM [4–7, 10–12], while increasing apoptosis [4, 8–10].

The conditional CreER-loxP deletion of β1 integrin in adult murine collagen I-producing cells (β1KO), one of the major ECM proteins in islets [13], demonstrated impaired glucose tolerance, decreased beta-cell mass and pancreatic and duodenal homeobox-1 (Pdx-1) expression, and a significant reduction in the phosphorylation of FAK and ERK1/2 signaling pathways [14]. Recently, a study using the *rat insulin 2* promoter Cre-loxP system (RIP-*Cre*) to induce a beta-cell specific knockout of β1 integrin (RIPβ1KO) found that β1 integrin is essential for maintaining beta-cell mass, but not function, during development and into adulthood [15]. However, the role of β1 integrin on beta-cell function and survival in a tightly regulated age-dependent manner has yet to be determined. Here, we utilize CreERT-loxP technology to generate a mouse model that uses the *mouse insulin 1* promoter (MIP) for the conditional knockout of beta-cell specific β1 integrin (MIPβ1KO), allowing us to determine the role of β1 integrin with respect to postnatal pancreatic beta-cell function and survival.

## RESULTS

### Characterization of β1 integrin expression in beta-cells of MIPβ1KO mice

**Figure 1:**
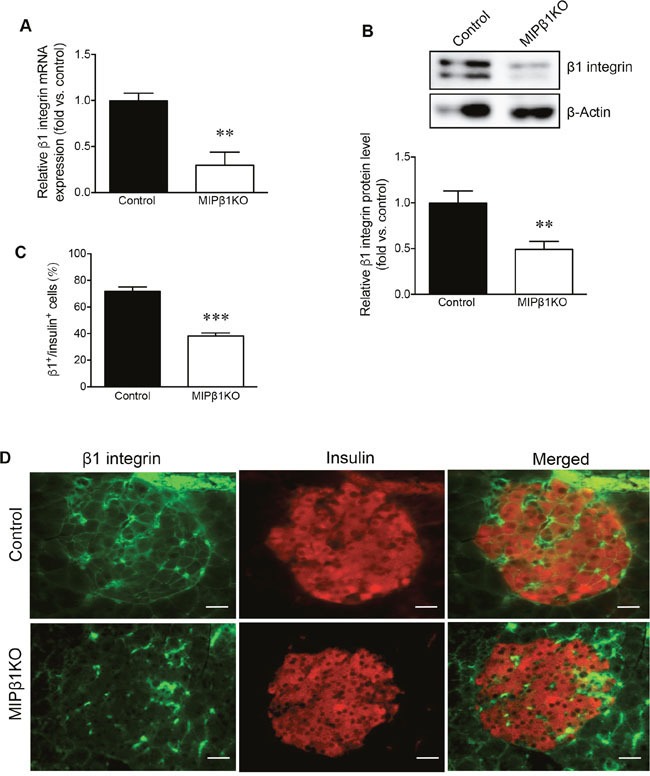
Confirmation of β1 integrin knockdown in MIPβ1KO mouse islets **(A)** qRT-PCR for *β1 integrin* mRNA and **(B)** western blot analysis for β1 integrin protein in male control and MIPβ1KO islets after 8 weeks post-tamoxifen, with a representative blot shown. Quantification **(C)** and representative images **(D)** of β1 integrin (green) and insulin (red) double immunofluorescence staining in the islets of control and MIPβ1KO mice. Scale bar: 25μm. Data are expressed as mean ± SEM *(n = 3-5/group)*. **p<0.01 ***p<0.001 vs. control group.

The level of β1 integrin knockdown in MIPβ1KO mouse islets was quantified. We observed that β1 integrin was significantly reduced at the mRNA and protein level (p<0.01 vs. control, Figure 1A-1B) by approximately 60% in male MIPβ1KO islets compared to controls at 8 weeks post-tamoxifen. Immunofluorescence staining confirmed a significant reduction of β1 integrin (∼45%) in the beta-cells of MIPβ1KO mice compared to control mice (p<0.001 vs. control, Figure 1C-1D).

### MIPβ1KO mice display impaired glucose tolerance

**Figure 2:**
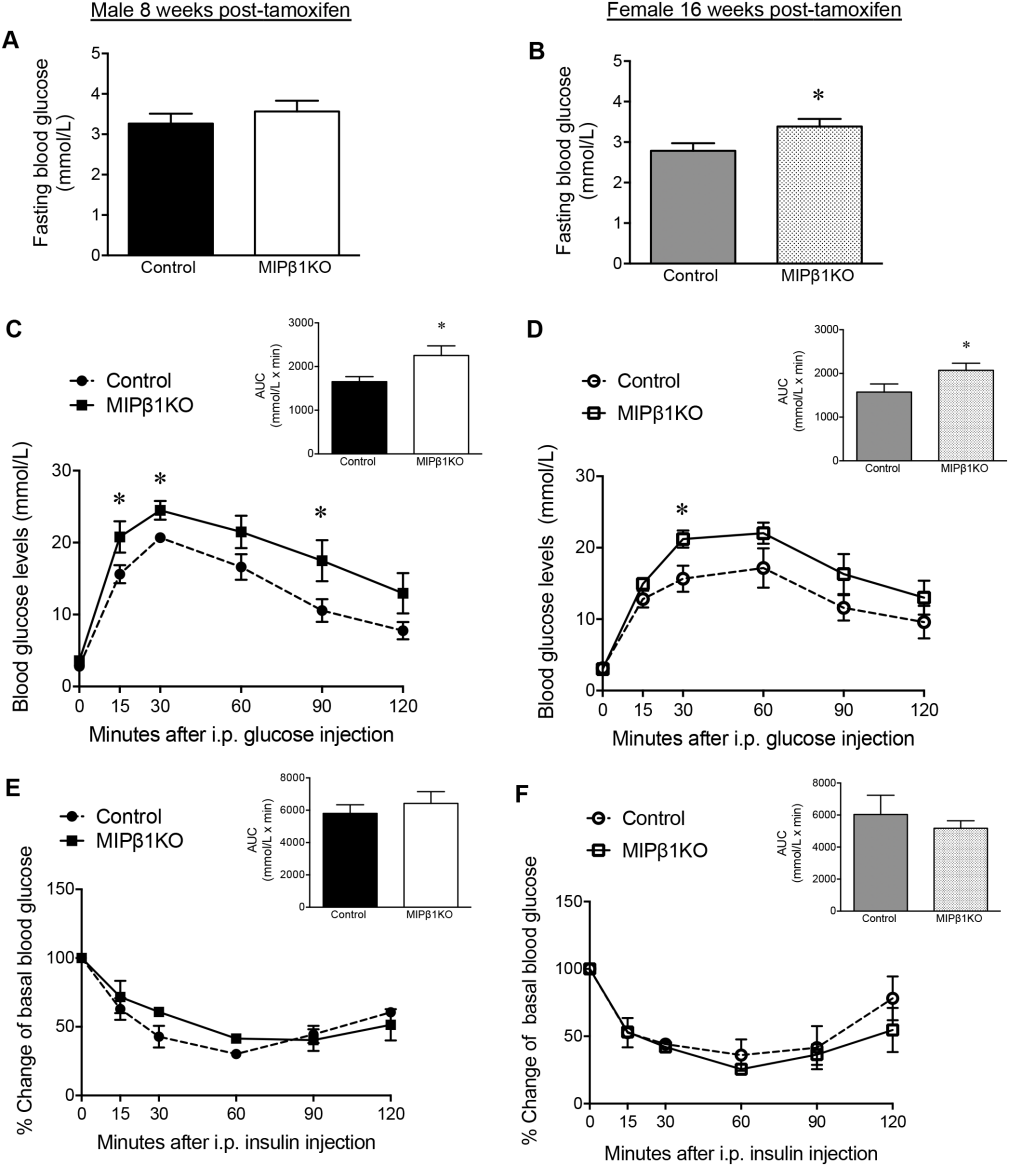
MIPβ1KO mice show impaired glucose metabolism Fasting blood glucose of control and MIPβ1KO mice after 8 (male, **A**) or 16 (female, **B**) weeks post-tamoxifen. IPGTT **(C, D)** and IPITT **(E, F)** analyses, and their corresponding AUC, for control and MIPβ1KO mice. Data are expressed as mean ± SEM (*n = 3-8/group*). *p<0.05 vs. control group.

Although no changes in fasting blood glucose were found in male MIPβ1KO mice 8 weeks post-tamoxifen (Figure 2A), 16 weeks post-tamoxifen female MIPβ1KO mice had significantly elevated fasting blood glucose levels compared to control littermates (p<0.05, Figure 2B). Body weight was unchanged in both male and female MIPβ1KO mice when compared to their same-sex littermates (data not shown). Male MIPβ1KO mice 8 weeks post-tamoxifen displayed impaired glucose tolerance as determined by an intraperitoneal glucose tolerance test (IPGTT) (p<0.05 vs. control, Figure 2C). Significantly impaired glucose tolerance was not observed in female MIPβ1KO mice until 16 weeks post-tamoxifen injection (p<0.05, Figure 2D). Intraperitoneal insulin tolerance tests (IPITT) revealed that both sexes of MIPβ1KO and control mice responded in a similar fashion at all time points (Figure 2E-2F).

### Deficient glucose-stimulated insulin secretion in male, but not female, MIPβ1KO mice

**Figure 3:**
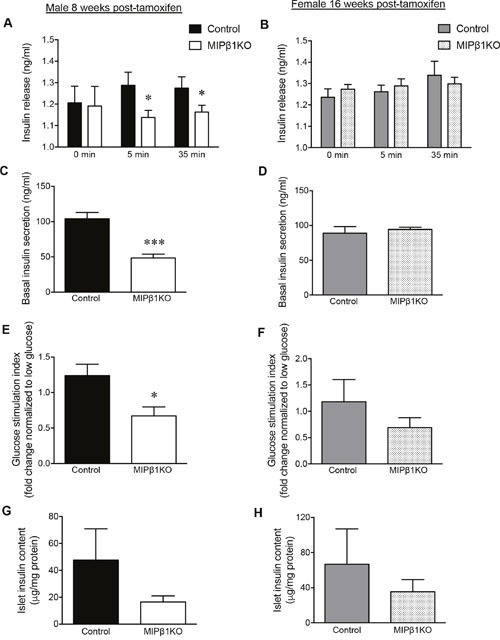
Impaired *in vivo* and *ex vivo* glucose-stimulated insulin secretion (GSIS) in male MIPβ1KO mice *In vivo* GSIS assay showing blood glucose and plasma insulin levels in male **(A)** and female **(B)** control and MIPβ1KO mice 8 or 16 weeks post-tamoxifen. Overnight basal insulin secretion from isolated islets of male **(C)** and female **(D)** control and MIPβ1KO mice. **(E, F)** *Ex vivo* insulin secretion in response to high glucose conditions displayed as fold change normalized to low glucose (2.2mM) secretion. Islet insulin content in isolated islets from male **(G)** and female **(H)** control and MIPβ1KO mice. Data are expressed as mean ± SEM (*n = 4-7/group*). *p<0.05; ***p<0.001 vs. control group.

Glucose-stimulated insulin secretion (GSIS) was measured *in vivo* and *ex vivo* to determine beta-cell functional responsiveness. *In vivo* GSIS results from male MIPβ1KO mice demonstrated lower levels of plasma insulin at 5 and 35 minutes post glucose injection when compared to littermate controls (p<0.05, Figure 3A). Basal insulin secretion from isolated male MIPβ1KO islets was reduced by ∼50% compared to control islets (p<0.001, Figure 3C), and *ex vivo* GSIS displayed reduced insulin secretion from male MIPβ1KO islets in response to glucose challenge (p<0.05, Figure 3E). Despite the impairment in glucose tolerance at 16 weeks post-tamoxifen, *in vivo* GSIS results from female MIPβ1KO mice showed similar levels of plasma insulin as controls (Figure 3B). Basal insulin secretion from isolated female MIPβ1KO islets was also unchanged (Figure 3D), and no significant reduction in insulin secretion was observed in *ex vivo* GSIS islets (Figure 3F). Islet insulin content of both male and female MIPβ1KO mice were reduced compared to control littermates (Figure 3G-3H), but statistical significance was not reached.

### Reduction of insulin secretory molecules in MIPβ1KO mice

**Figure 4:**
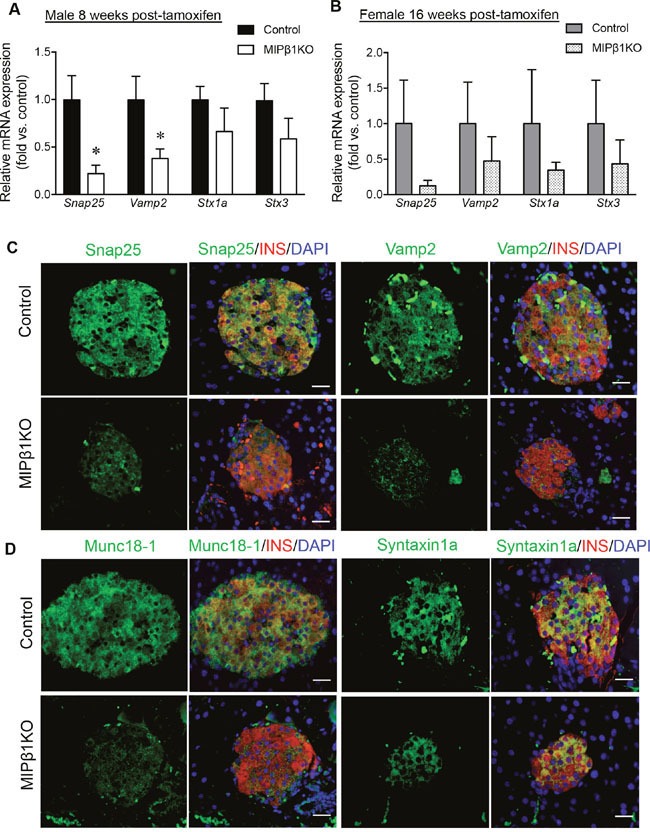
Reduced insulin exocytotic machinery in MIPβ1KO mice Relative mRNA expression of *Snap25*, *Vamp2*, *Stx1a* and *Stx3* in male **(A)** and female **(B)** control and MIPβ1KO mice 8 or 16 weeks post-tamoxifen. Data are expressed as mean ± SEM *(n = 3-4/group*). *p<0.05 vs. control. **(C, D)** Representative immunofluorescence staining images for Snap25, Vamp2, Munc18-1 and Syntaxin1a (green) with insulin (red) in male control and MIPβ1KO mice. Nuclei were labeled using DAPI (blue) (*n=3/group*). Scale bar: 25μm.

To determine whether impaired GSIS is due to a defect in the exocytosis of insulin granules from beta-cells, the exocytotic proteins involved in SNARE (Soluble N-Ethylmaleimide Sensitive-Factor Attachment Protein Receptor) complex formation were examined. A significant reduction in *synaptosome associated protein 25kDa* (*Snap25*) and *vesicle-associated membrane protein 2* (*Vamp2*) (p<0.05) mRNA, with relatively low levels of *syntaxin 1a* (*Stx1a*) and *syntaxin 3* (*Stx3*) mRNA, was observed in male MIPβ1KO mice compared to controls (Figure 4A). Immunofluorescence staining showed a clear reduction in beta-cell Snap25 and Vamp2 protein levels, in alignment with the reduction in mRNA observed in male MIPβ1KO mice (Figure 4C). Mammalian uncoordinated-18 (Munc18-1) was also diminished in the beta-cells of male MIPβ1KO mice with no change in syntaxin 1a staining (Figure 4D). Female MIPβ1KO mice displayed a similar trend with reduced *Snap25, Vamp2, Stx1a* and *Stx3* mRNA, but the results were not statistically significant compared to controls (Figure 4B).

### Reduced Pdx-1 levels in male, but not female, MIPβ1KO mice

**Figure 5:**
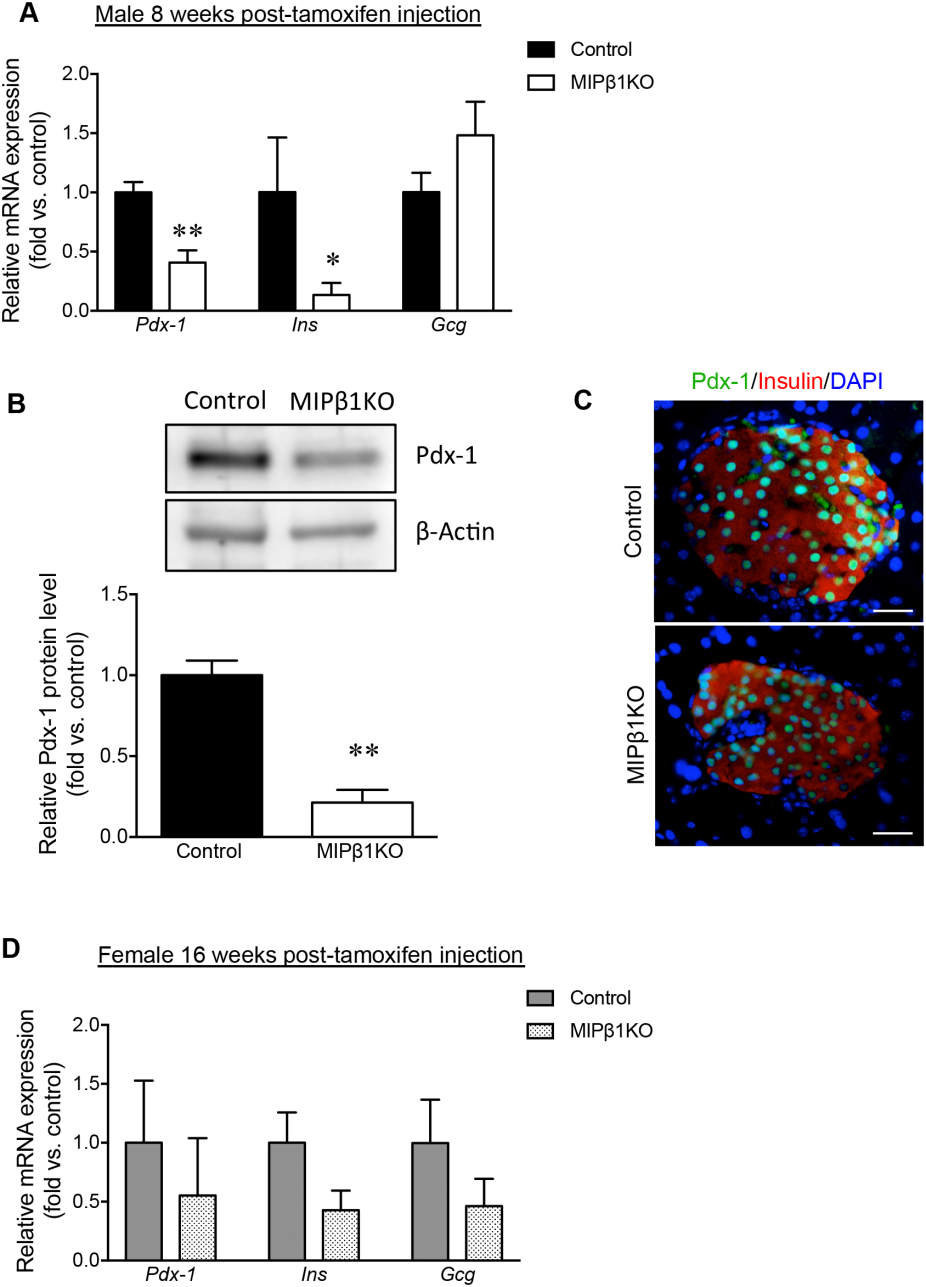
Decreased Pdx-1, insulin, and glucagon expression in MIPβ1KO mice **(A)** qRT-PCR analysis for *Pdx-1, Ins*, and *Gcg* mRNA expression in male control and MIPβ1KO mice 8 weeks post-tamoxifen. **(B)** Pdx-1 protein levels in male control and MIPβ1KO mouse islets, with representative blot shown. **(C)** Representative immunofluorescence images for Pdx-1 (green), insulin (red) and DAPI (blue) in male control and MIPβ1KO mouse islets. Scale bar: 25μm. **(D)** qRT-PCR analysis for *Pdx-1, Ins*, and *Gcg* mRNA expression of female control and MIPβ1KO mice at 16 weeks post-tamoxifen. Data are expressed as mean ± SEM (*n=3-6/group*). *p<0.05, **p<0.01 vs. control group.

Pdx-1 is an important regulator of insulin secretion [16], and both mRNA and protein levels were significantly reduced in the previously reported β1KO model [14]. Male MIPβ1KO mice display a 40% reduction of *Pdx-1* mRNA in islets when compared to control mice (p<0.01, Figure 5A), along with a significant reduction in Pdx-1 protein level as measured by western blot (p<0.01, Figure 5B). Immunofluorescence staining for Pdx-1 in beta-cells of male MIPβ1KO mice was consistently less intense than in controls (Figure 5C). We further examined additional transcription factors involved in postnatal beta-cell regulation and function (NK6 homeobox 1 (Nkx6.1), NK2 homeobox 2 (Nkx2.2), Islet-1 (Isl-1), and v-maf avian musculoaponeurotic fibrosarcoma homolog A (MafA)), and found similar staining intensities between male MIPβ1KO mice and control mice (Supplementary Figure 1). A significant reduction of *Insulin*, but not *Glucagon*, mRNA in male MIPβ1KO mouse islets was also detected (Figure 5A). The mRNA levels of *Pdx-1*, *Insulin*, and *Glucagon* in female MIPβ1KO mouse islets were relatively lower than their control littermates, but did not reach statistical significance (Figure 5D).

### Reduction of beta-cell mass in MIPβ1KO mice, but no alteration in islet vasculature

**Figure 6:**
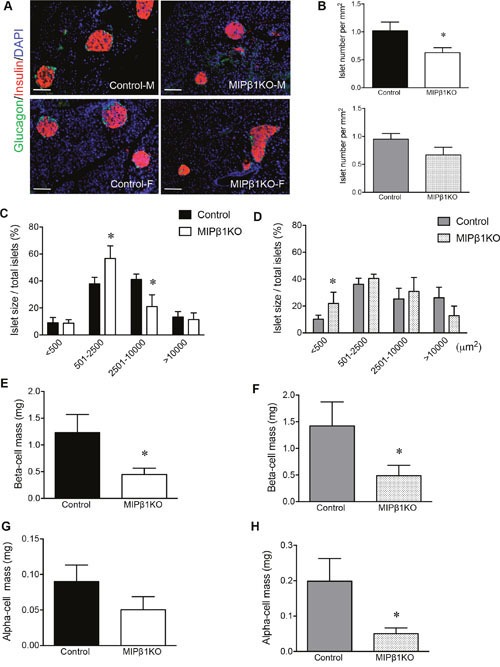
MIPβ1KO mice display smaller islets with loss of beta-cell mass **(A)** Representative immunofluorescence staining of glucagon (green) and insulin (red) to indicate islet labeling. Nuclei were stained with DAPI (blue) in both male and female control and MIPβ1KO mice. Scale bar: 200μm. Islet number **(B)**, islet size **(C, D)**, beta-cell mass **(E, F)** and alpha-cell mass **(G, H)** of control and MIPβ1KO mice 8 or 16 weeks post-tamoxifen. Data are expressed as mean ± SEM (*n=5-6/group*). *p<0.05 vs. control group.

Immunofluorescence staining of MIPβ1KO and control pancreata showed that the overall architecture of the pancreas was unaltered, with intact islets and a normal distribution of insulin^+^ and glucagon^+^ cells observed (Figure 6A). Islet density (islets per mm^2^) was significantly reduced in male MIPβ1KO pancreata (p<0.05), while relatively lower islet density was observed in female MIPβ1KO pancreata compared to the controls (Figure 6B). Male MIPβ1KO mice demonstrated a significantly higher percentage of smaller islets (<2,500μm^2^) and a lower number of large islets (<10,000μm^2^; p<0.05, Figure 6C). Female MIPβ1KO mice also showed a higher percentage of the smallest islets (<500μm^2^) and a low number of the largest islets (>10,000μm^2^) when compared to control mice (p<0.05, Figure 6D). The decrease in larger islets was accompanied by significantly reduced beta-cell mass in both male and female MIPβ1KO mice (p<0.05, Figure 6E-6F). Reductions in alpha-cell mass were observed in female (p<0.05, Figure 6H), but not male (Figure 6G), MIPβ1KO pancreata.

Immunofluorescence staining for E-cadherin and Glut2, proteins required for the maintenance of beta-cell function, displayed no change in male or female MIPβ1KO islets (Supplementary Figure 2). Additional integrin subunits were also examined within islets of male MIPβ1KO mice. qRT-PCR analyses of *α6* and *αV integrin* mRNA were relatively lower than that of controls, while *α3* and *α5 integrin* mRNA were unchanged (Supplementary Figure 3A). Lower α6 and αV, but increased α3 integrin staining intensity was observed in male MIPβ1KO islets compared to controls (Supplementary Figure 3B). In contrast, α5 integrin, which is predominantly expressed in alpha-cells, showed no change in staining intensity (Supplementary Figure 3B).

Although male β1KO mice had a significant reduction in islet capillary area [14], qualitative assessment of PECAM^+^ blood vessel area and diameter in MIPβ1KO mouse islets showed no obvious alterations in either gender of MIPβ1KO mouse islets at 8 (male) or 16 weeks (female) post-tamoxifen when compared to controls (Supplementary Figure 4).

### Reduction of phosphorylated-FAK, ERK1/2, and Akt protein levels with reduced cell proliferation in male MIPβ1KO mice

**Figure 7:**
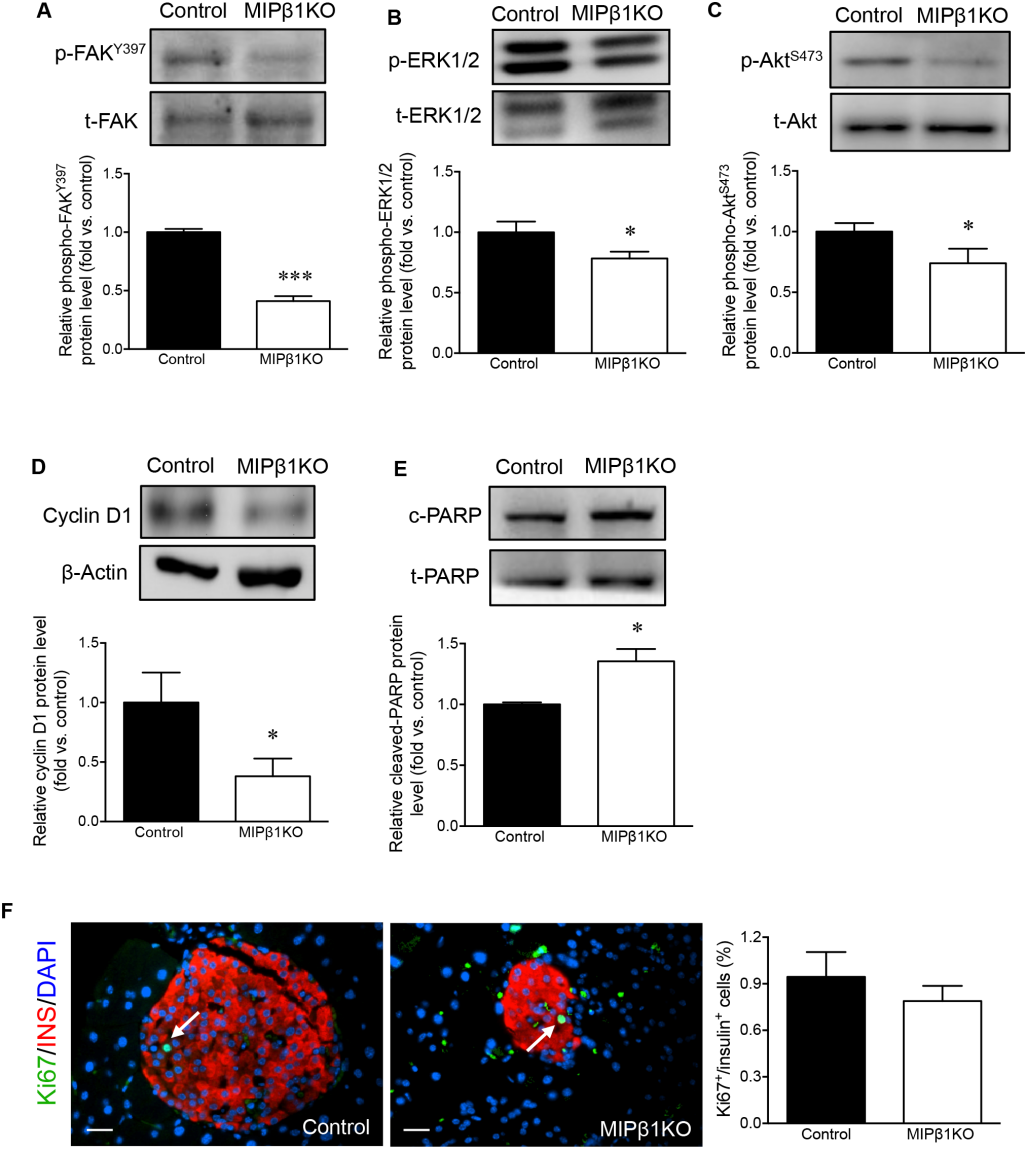
Altered intracellular cell signaling, proliferation, and apoptotic protein levels in male MIPβ1KO mouse islets Western blot analyses for p-FAK^Y397^ **(A)**, pERK1/2 **(B)**, p-Akt^S473^ **(C)**, cyclin D1 **(D)** and c-PARP **(E)** protein levels with representative blots shown for male control and MIPβ1KO mouse islets 8 weeks post-tamoxifen. **(F)** Representative immunofluorescence staining for Ki67 (green), insulin (red), and DAPI (blue in nuclei), and quantification of Ki67^+^ beta-cells. White arrows indicate nuclear localization of Ki67 in insulin^+^ cells. Scale bar: 25μm. Data are expressed as mean ± SEM (*n = 3-11/group*). *p<0.05, ***p<0.001 vs control.

FAK, ERK1/2, and Akt are downstream signaling molecules of β1 integrin that have been shown to regulate beta-cell proliferation, survival, and function [4, 12, 14, 17]. There was a significant reduction in p-FAK^Y397^ (p<0.001, Figure 7A) and p-ERK1/2 (p<0.05, Figure 7B) in male MIPβ1KO mouse islets compared to controls. A significant decrease in p-Akt^S473^ was also observed in male MIPβ1KO islets (p<0.05, Figure 7C). Protein levels of the proliferative marker cyclin D1 were found to be significantly reduced in male MIPβ1KO mouse islets compared to controls (p<0.05, Figure 7D), and a significant increase in the apoptotic marker cleaved-Poly (ADP-ribose) polymerase (c-PARP) was also evident in male MIPβ1KO islets (p<0.05, Figure 7E). The percentage of Ki67^+^ beta-cells in male MIPβ1KO islets was also relatively lower than that of controls (Figure 7F).

### Glucose intolerance was maintained in aged MIPβ1KO mice

**Figure 8:**
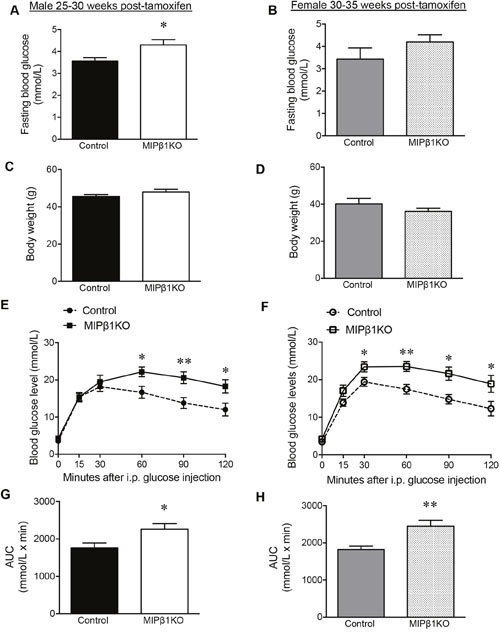
Impairment of glucose metabolism in aged MIPβ1KO mice Fasting blood glucose and body weight of aged male **(A, C)** and female **(B, D)** control and MIPβ1KO mice. IPGTT analysis of aged control and MIPβ1KO male **(E)** and female **(F)** mice, and corresponding AUC **(G, H)**. Data are expressed as mean ± SEM (*n=6-14/group*). *p<0.05, **p<0.01 vs. control.

To investigate whether MIPβ1KO mice maintained long-term glucose intolerance, both male and female MIPβ1KO mice were examined at 25-35 weeks post-tamoxifen. The aged MIPβ1KO mice maintained the reduction of β1 integrin levels (∼40% loss) in beta-cells when compared to controls (Supplementary Figure 5). Aged male MIPβ1KO mice showed a significant elevation in overnight fasting blood glucose levels (p<0.05, Figure 8A). Interestingly, aged female MIPβ1KO mice also developed higher fasting blood glucose levels (Figure 8B). Similar body weight was observed in all aged groups (Figure 8C-8D). IPGTT in aged male MIPβ1KO mice showed significantly elevated glucose levels at 60, 90, and 120 minutes after glucose injection (p<0.05-0.01, Figure 8E), and an overall increase in AUC when compared to control mice (p<0.05, Figure 8G). Aged female MIPβ1KO mice also demonstrated significantly increased glucose levels (30, 60, 90, and 120 minutes post glucose load) (p<0.05-0.01, Figure 8F) and AUC (p<0.01, Figure 8H) during an IPGTT.

## DISCUSSION

The inducible MIPβ1KO mouse model allowed for *in vivo* evaluation of beta-cell-specific β1 integrin activity during postnatal islet function. A significant reduction of beta-cell β1 integrin expression in the adult mouse resulted in impaired glucose tolerance and insulin secretion, reduced beta-cell mass, decreased signaling through the FAK/ERK and Akt pathways, and diminished beta-cell function and expansion. This study further demonstrates that β1 integrin in adult pancreatic beta-cells is essential for maintaining normal beta-cell mass and insulin secretion.

In contrast to previous studies that utilized a global β1KO model [14, 18], there was no difference in pancreatic weight and macrostructure between MIPβ1KO and control mice, indicating that the targeted loss of β1 integrin in beta-cells does not compromise the architecture of the pancreas. The impaired glucose tolerance displayed in male MIPβ1KO mice at 8 weeks post-tamoxifen administration is similar to postnatal results from global β1KO mice and beta-cell specific FAK-knockout mice [14, 19]. However, this observation is not in alignment with the RIPβ1KO study [15], which found normal glucose levels when β1 integrin was knocked out in beta-cells at conception. Furthermore, an increase in ECM components vitronectin and laminin-5 β-chain, which are ligands of αVβ3 and α6β4 respectively, was observed in RIPβ1KO islets. This result suggests a possible compensatory response during embryonic development and postnatal remodelling that might be responsible for maintained glucose homeostasis. We found a reduction of αV and α6 integrin mRNA and protein in MIPβ1KO islets, yet aged MIPβ1KO mice (25-35 weeks post-tamoxifen) maintained impaired beta-cell function, suggesting that there was no compensatory effect from other integrins after postnatal knockdown of β1 integrin.

Examination of the exocytotic proteins involved in insulin secretion showed reduced Vamp2 and Snap25 mRNA and protein levels in male MIPβ1KO mice, which impairs docking and release of insulin vesicles from beta-cells [20]. It has been shown that focal adhesion remodelling is required for GSIS through FAK-paxillin interactions, which subsequently increase the levels of activated Snap25 [19, 21]. β1 integrin is an essential component for focal adhesion formation and its loss appears to alter the insulin exocytotic machinery through impaired downstream focal adhesion signaling. Although no significant changes in Stx1a and Stx3 were found, a reduction in Munc18-1 protein in the beta-cells of MIPβ1KO mice was observed. Munc18-1 is a syntaxin binding protein that plays a dual role in transporting Stx1a to the plasma membrane and regulating SNARE mediated vesicle fusion [22–24]. Furthermore, Pdx-1, a transcription factor required for proper maintenance and function of beta-cells in adult mice [16], showed a marked decrease in mRNA and protein in male MIPβ1KO mouse islets. Reduced Pdx-1 could contribute to impaired beta-cell function, insufficient insulin production, and the reduction in beta-cell mass that was observed in male MIPβ1KO mice. Taken together, these observations show that β1 integrin in beta-cells is required for maintaining normal physiological levels of exocytotic machinery, which are pivotal in insulin release.

Our study found a significant reduction in the phosphorylation of FAK^Y397^, a key component in transducing ‘outside-in’ signaling through β1 integrin and responsible for activating multiple downstream signaling molecules [14]. One protein downstream of FAK that was reduced in MIPβ1KO mouse islets was ERK1/2, important in maintaining function and survival of beta-cells and beta-like cells *in vitro* [4, 9, 12, 25], and beta-cells *in vivo* [13, 14]. Activation of ERK has been shown to play a role in not only beta-cell survival, but also in potentially regulating Pdx-1 expression [4, 9, 12, 14] and GSIS through actin remodelling [21, 25]. FAK has also been shown to mediate insulin signaling through activation of Akt [19]. Similar to RIPβ1KO mice [15] and the beta-cell specific FAK knockdown mouse line [19], MIPβ1KO mice also had decreased phosphorylated Akt^S473^. Akin to our previous study [14], a significant reduction in proliferation (cyclin D1) and an increase in apoptosis (c-PARP) were observed in male MIPβ1KO mice. These findings indicate that β1 integrin signals primarily through the FAK/ERK and Akt cascade *in vivo* to regulate glucose metabolism, beta-cell survival and function.

Male mice with a global β1KO [14, 18] displayed severely compromised pancreas architecture and beta-cell function compared to their female littermates. Female MIPβ1KO mice showed no significant impairment in glucose tolerance until 16 weeks post-tamoxifen, demonstrating that there are gender differences in glucose metabolism and an underlying protective factor in female mice. Estrogen administration has been shown to protect mice from beta-cell ablation in streptozotocin and alloxan-induced diabetes by preventing beta-cell apoptosis [26–27]. Despite only seeing a significant change in proliferative and apoptotic markers in male MIPβ1KO mice, a significant reduction in both alpha- and beta-cell mass was observed in female MIPβ1KO mice. In a model of diet-induced diabetes, like in the Goto-Kakizaki rat [28], alpha-cell mass can decrease when beta-cells are reduced. The defect in alpha-cell mass could be attributed to a reduced beta-cell population, which subsequently impacts cell-to-cell contact within the islet, along with reduced nutrient secretion and protein secretion important in eliciting normal ECM development in and around the islets.

In summary, this study is the first to utilize a temporal knockout of β1 integrin in beta-cells during the postnatal period and demonstrates that diminished β1 integrin leads to compromised beta-cell function and survival through decreased FAK/ERK and Akt signaling cascades. Reduced β1 integrin also leads to a decrease in SNARE proteins Snap25, Vamp2, and Munc18-1, indicating β1 integrin is important for their activation during insulin exocytosis. Understanding how β1 integrin maintains postnatal beta-cell insulin secretion and survival could aid in the development of tissue engineering strategies for a bioartificial endocrine pancreas, a new strategy for cell-based therapeutic approaches for the treatment of both type 1 and type 2 diabetes.

## MATERIALS AND METHODS

### Inducible beta-cell specific β1 integrin -deficient mouse model

*B6;129-Itgb1^tm1Efu/J^* (*β1^fl/fl^*) and *Tg(Ins1-Cre/ERT)^1Lphi^* (*MIP-CreERT*) mice were obtained from Jackson Laboratories (Bar Harbor, MA, USA; stock number: 004605) and Dr. Louis Philipson's laboratory (University of Chicago, Chicago, IL, USA) [29], respectively. *MIP-CreERT* and *β1^fl/fl^* mice were crossed to produce *MIP-CreERT^+/−^;β1itg^fl/+^* mice, and these mice were mated to establish the tamoxifen inducible β1 integrin knockout experimental mouse model. Tamoxifen (Sigma, St. Louis, MO, USA) administration began at 4 weeks of age and was injected intraperitoneally for 3 consecutive days at a dose of 4mg/20g bodyweight to all mice used in this study. Mice were genotyped for β1 integrin and MIP-CreERT expression using primers listed in Supplementary Table 1. Experimental *MIP-CreERT^+^*;*β1itg^fl/fl^* (**MIPβ1KO**) mice are MIP-CreERT positive (268bp) with both β1 integrin alleles floxed by LoxP sites (β*1itg^fl/fl^*, 280bp). *MIP-CreERT-; β1itg^fl/fl^* and *MIP-CreERT^+;^β1itg^+/+^* (**Controls)** mice are either MIP-CreERT negative with both β1 integrin alleles floxed or MIP-CreERT positive with wild-type β1 integrin alleles (*β1itg^+/+^*, 160bp) [30]. All protocols were approved by the Animal Use Subcommittee at the University of Western Ontario in accordance with the guidelines of the Canadian Council of Animal Care.

### Glucose metabolic studies and glucose-stimulated insulin secretion

Intraperitoneal glucose and insulin tolerance tests (IPGTT and IPITT, respectively) were conducted at 8, 16, and 25-35 weeks post tamoxifen injection in MIPβ1KO and control mice. For IPGTT, glucose (D-(+)-glucose; dextrose; Sigma-Aldrich Canada Co., Oakville, Ontario, Canada) was administered intraperitoneally at a dose of 2mg/g of bodyweight after a 16 hour fast, and blood glucose levels were recorded over a 120 minute duration. Insulin (Humalin, Eli Lilly, Toronto, Ontario, Canada) at 1 U/kg of body weight was injected intraperitoneally after a 4 hour fast for IPITT. Area under the curve (AUC) was used to quantify glucose or insulin responsiveness using units of mmol/L x minute. GSIS tests were performed at 8 and 16 weeks post-tamoxifen, and blood samples were collected via tail vein at baseline insulin levels (0 minutes), and at 5 and 35 minutes after glucose loading [14].

For *ex vivo* GSIS, islets from both MIPβ1KO and control mice at 8 and 16 week post-tamoxifen were isolated and hand-picked with 10 islets in duplicate per experimental group. Isolated islets were recovered in RPMI 1640 plus 10% FBS overnight. Islets were incubated for one hour in low - high - low (2mM - 22mM - 2mM) serum-free glucose media. The stimulation media was collected for insulin release measurements and islets were collected for insulin content analysis [31].

Plasma insulin, basal insulin release, and insulin secretion under glucose-stimulation were measured using the Mouse Ultrasensitive Insulin ELISA (ALPCO®). A static glucose stimulated insulin secretion index was calculated by dividing the insulin output from the high glucose (22 mM) incubation by the insulin output during the low glucose (2.2 mM) incubation [31]. Islet insulin content was measured and expressed as micrograms per milligram of protein [31].

### Immunofluorescence and morphometric analyses

Pancreata from MIPβ1KO and control mice at 8, 16, or 25-35 weeks post-tamoxifen were fixed in 4% paraformaldehyde and embedded in paraffin. Pancreatic sections were prepared from the entire length of the pancreas and stained with primary antibodies listed in Supplementary Table 2. Quantitative evaluations of islet number, size, and alpha-cell and beta-cell mass were performed using Image-Pro software (MediaCybernetics, Rockville, MD, USA) [14, 30]. A minimum of 10 islets per pancreas was manually traced from 5 to 6 different mice per experimental group. The percentages of Pdx-1, Nkx6.1, and Ki67 co-localization in insulin^+^ cells were determined by cell counting using double immunofluorescence staining. Islet capillary density and diameter were measured using PECAM^+^ identification, and data are expressed as a percentage [14, 17].

### RNA extraction and real-time RT-PCR

RNA was extracted from isolated islets of MIPβ1KO and control mice using the RNAqueous-4PCR kit (Invitrogen) [16]. Sequences of PCR primers are provided in Supplementary Table 3. Real-time PCR analyses were performed using the iQ SYBR Green Supermix kit (Bio-Rad Laboratories, Mississauga, ON, Canada). Relative levels of gene expression were calculated and normalized to the internal gene 18S rRNA, with at least three biological repeats per experimental group [17].

### Protein extraction and western blot analysis

Isolated islets were sonicated and extracted in a Nonidet-P40 lysis buffer (Sigma-Aldrich; St Louis, MO, USA). Proteins were separated via electrophoresis on either a 5%, 7.5%, or 10% sodium dodecyl sulfate-polyacrylamide gel and transferred onto a nitrocellulose membrane (Bio-Rad Laboratories, Hercules, CA, USA). Primary antibodies used for detection are listed in Supplementary Table 2. Proteins were detected using ECL™-Plus Western Blot detecting reagents (Perkin-Elmer, Wellesley, MA, USA) and imaged using the Versadoc Imaging System (Bio-Rad Laboratories). Image Lab software (Bio-Rad Laboratories) was used to quantify band intensities using densitometry, and data were normalized to total or appropriate loading controls [17, 30, 31].

### Statistical analyses

Data are expressed as means ± SEM. Statistical significance was determined using the unpaired student's *t*-test. Differences were considered statistically significant when *p*<0.05.

## SUPPLEMENTARY MATERIALS FIGURES AND TABLES


