# Supplementary material for: Critical role of β1 integrin in postnatal beta-cell function and expansion
Source: Oncotarget. 2017 May 18;8(38):62939–52. doi: 10.18632/oncotarget.17969 (PMC5609893; doi:10.18632/oncotarget.17969)
Supplement: Supplementary file 1 [file oncotarget-08-62939-s001.pdf]

Critical role of β1 integrin in postnatal beta-cell function and expansion

SUPPLEMENTARY MATERIALS

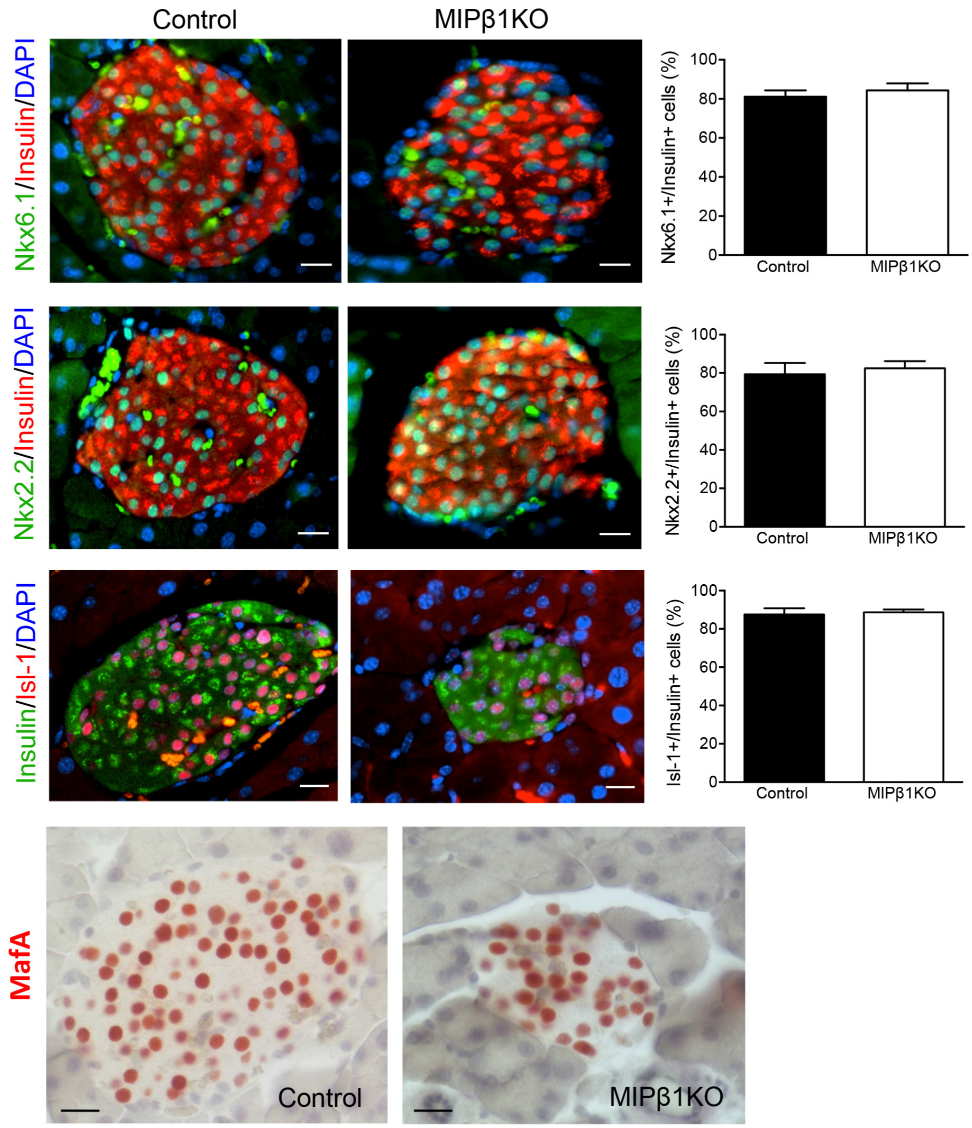

Supplementary Figure 1: Transcription factor expression in 8 week post-tamoxifen male MIPβ1KO mice. No quantitative changes were noted between groups for Nkx6.1, Nkx2.2, Isl-1, and MafA in control and MIPβ1KO mice, as determined by dual immunofluorescence staining or immunohistochemical staining. Data are expressed as mean ± SEM (*n = 2-5/group*). Scale bar: 25 μm.

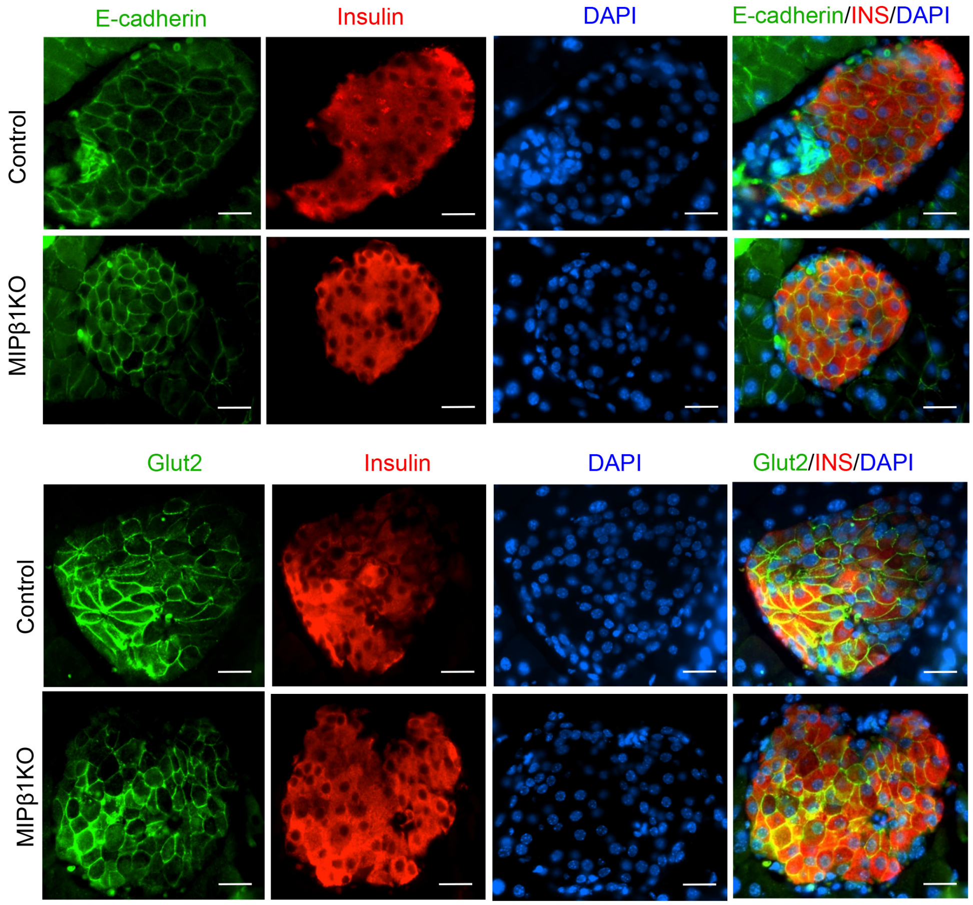

Supplementary Figure 2: E-cadherin and Glut2 co-localization in 8 week post-tamoxifen male MIPβ1KO mice. The islets of control and MIPβ1KO mice show no changes between the two groups for cell adhesion molecule e-cadherin (above) and glucose-sensing transporter Glut2 (below). Scale bar: 25 µm.

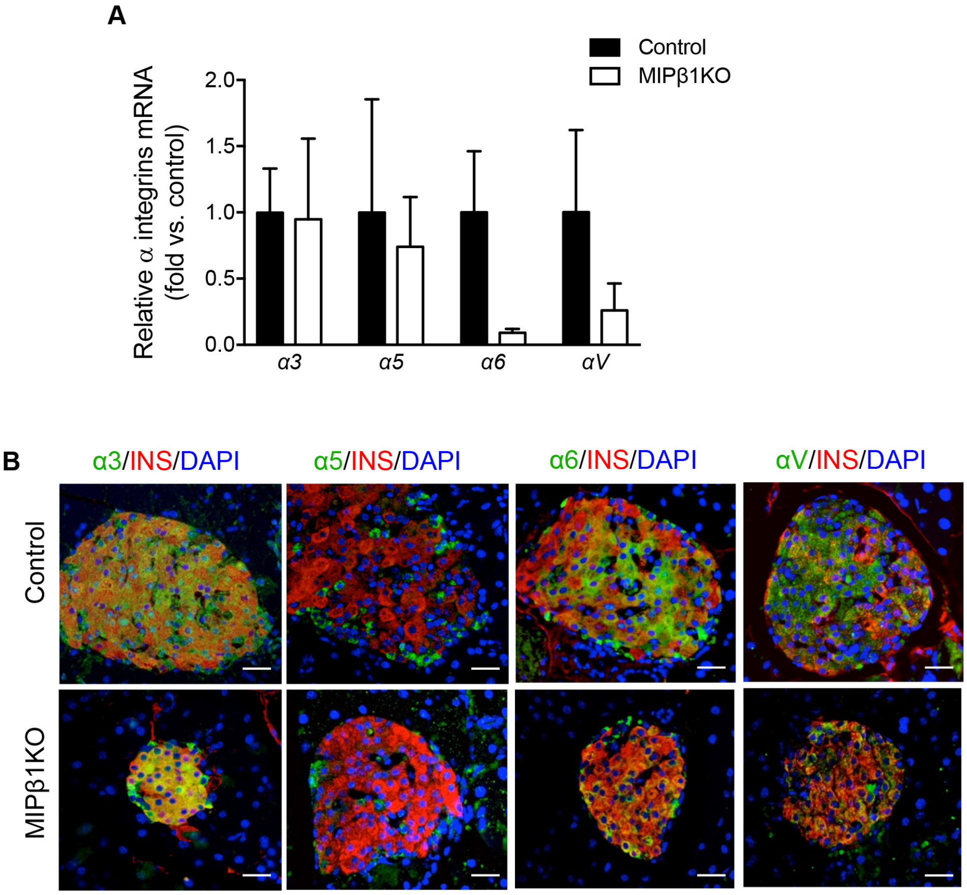

Supplementary Figure 3: Analyses for integrin alpha subunits in 8 week post tamoxifen male MIPβ1KO mice. (A) Relative mRNA expression for: *α3*, *α5, α6* and *αV* integrin in control and MIPβ1KO mouse islets. Data are expressed as mean ± SEM (*n = 3-4/group*). **(B)** Representative immunofluorescence images for α3, α5, α6 and αV integrin (green) with insulin (red) in control and MIPβ1KO mice. Nuclei stained with DAPI (blue) (*n=3/group*). Scale bar: 25 µm.

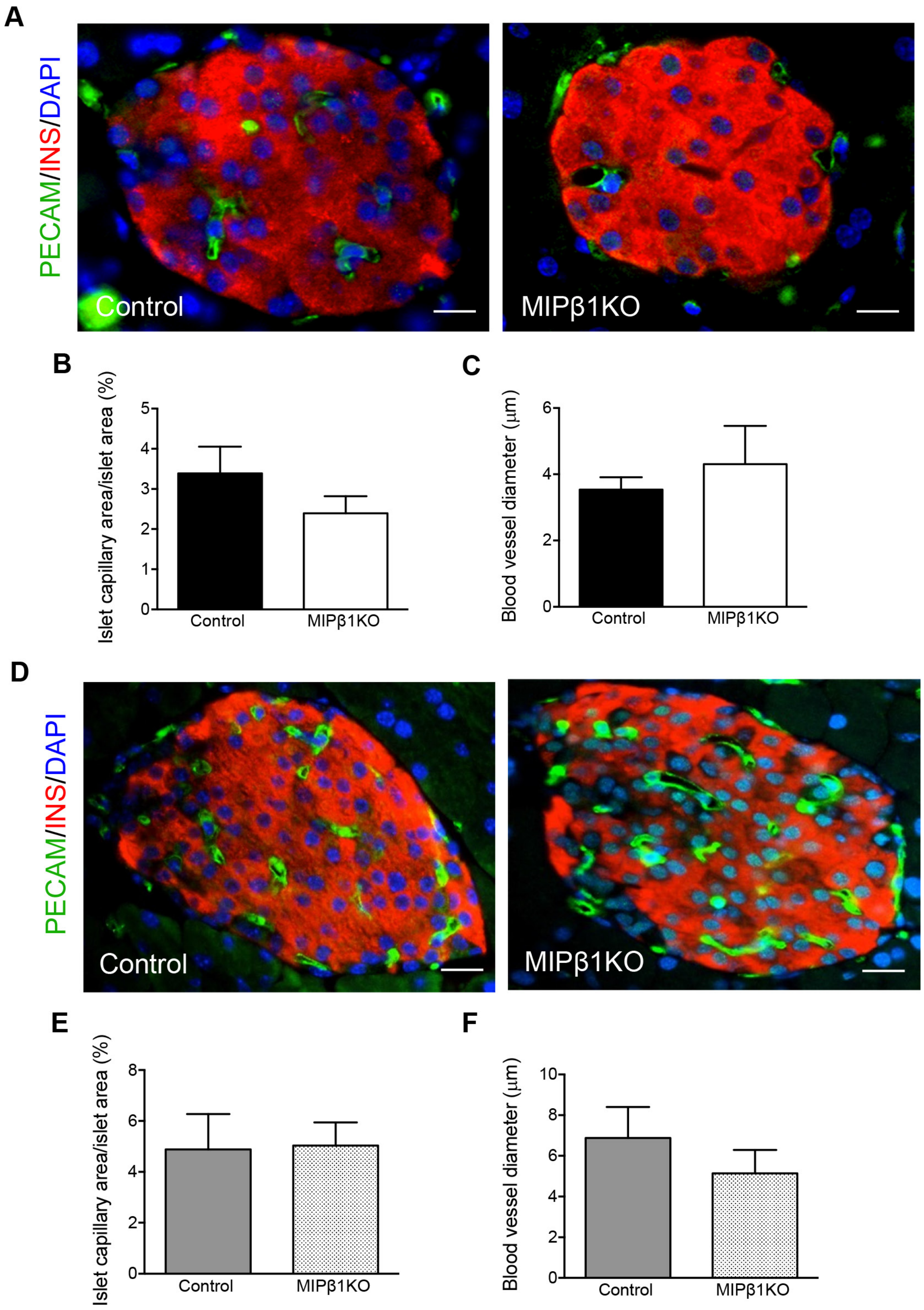

Supplementary Figure 4: Measurement of islet vascularization in MIPβ1KO mice. Representative immunofluorescence staining of PECAM (green), insulin (red) and DAPI (blue) in male **(A)** and female **(D)** control and MIPβ1KO mice. Scale bar: 25 μm. Quantification of blood vessel area and diameterin male **(B, C)** and female **(E, F)** control and MIPβ1KO mice. Data are expressed as mean ± SEM (*n = 2-5/group*).

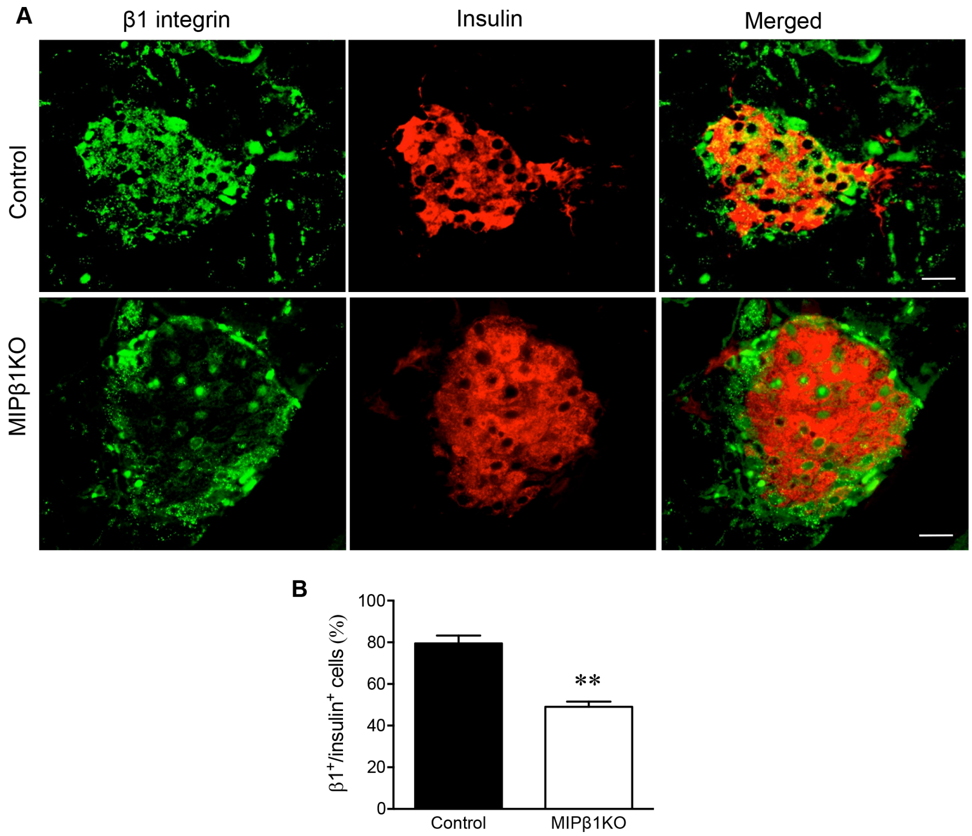

Supplementary Figure 5: Confirmation of β1 integrin knockdown in aged MIPβ1KO mouse islets. **(A)** Representative double immunofluorescence staining for β1 integrin (green) and insulin (red) in the islets of control and MIPβ1KO male mice after 25-30 weeks post-tamoxifen. Scale bar: 25μm. **(B)** Percentage of beta-cells that stained positive for β1 integrin. Data are expressed as mean ± SEM *(n = 3/group)*. ***p<0.01* vs. control group.

Supplementary Table 1: List of primers used for polymerase chain reactions

Primer name	Primer pair sequence
oIMR1906	5′-CGG CTC AAA GCA GAG TGT CAG TC-3′
oIMR1907	5′-CCA CAA CTT TCC CAG TTA GCT CTC-3′
MIP	5′- CCT GGC GAT CCC TGA ACA TGT CCT - 3′
CreER	5′- TGG ACT ATA AAG CTG GTG GGC AT - 3′

Supplementary Table 2: List of antibodies used for immunostaining and western blot analyses

Primary antibodies		Dilution	Company
Anti-Akt	Rabbit polyclonal	1:3000*	Cell Signaling (Temecula, CA, USA)
Anti-Cyclin D1	Mouse monoclonal	1:2000*	Cell Signaling (Temecula, CA, USA)
Anti-E-Cadherin	Rabbit polyclonal	1:200	Cell Signaling (Temecula, CA, USA)
Anti-ERK	Rabbit polyclonal	1:3000*	Cell Signaling (Temecula, CA, USA)
Anti-FAK	Rabbit polyclonal	1:1000*	Cell Signaling (Temecula, CA, USA)
Anti-Glucagon	Rabbit polyclonal	1:800	Sigma-Aldrich (St Louis, MO, USA)
Anti-Glut2	Rabbit polyclonal	1:200	Abcam (Cambridge, MA, USA)
Anti-Insulin	Mouse monoclonal	1:800	Sigma-Aldrich (St Louis, MO, USA)
Anti-Insulin	Rabbit polyclonal	1:50	Santa Cruz (Santa Cruz, CA, USA)
Anti-Integrin α3	Rabbit polyclonal	1:50	Chemicon (Temecula, CA, USA)
Anti-Integrin α5	Rabbit polyclonal	1:50	Chemicon (Temecula, CA, USA)
Anti-Integrin α6	Rabbit polyclonal	1:50	Santa Cruz (Santa Cruz, CA, USA)
Anti-Integrin αV	Rabbit polyclonal	1:100	Abcam (Cambridge, MA, USA)
Anti-Integrin β1	Rabbit polyclonal	1:500*	Abcam (Cambridge, MA, USA)
Anti-Integrin β1	Rat monoclonal	1:100	Milipore (Etobicoke, ON, CA)
Anti-Islet1	Mouse monoclonal	1:100	DSHB (University of Iowa, Iowa City, USA)
Anti-Ki67	Rabbit polyclonal	1:100	Abcam (Cambridge, MA, USA)
Anti-MafA	Rabbit polyclonal	1:100	Bethyl Laboratory (Montgomery, TX, USA)
Anti-Munc18-1	Rabbit polyclonal	1:100	Abcam (Cambridge, MA, USA)
Anti-Nkx2.2	Mouse monoclonal	1:100	DSHB (University of Iowa, Iowa City, USA)
Anti-Nkx6.1	Mouse monoclonal	1:100	DSHB (University of Iowa, Iowa City, USA)
Anti-PARP	Rabbit polyclonal	1:1000*	Cell Signaling (Temecula, CA, USA)
Anti-Pdx-1	Rabbit polyclonal	1:800<br>1:2000*	Dr. Wright (University of Vanderbilt, Nashville, TN, USA)
Anti-PECAM	Rabbit polyclonal	1:200	Santa Cruz (Santa Cruz, CA, USA)
Anti-phospho-Akt (Ser473)		1:2000*	Cell Signaling (Temecula, CA, USA)
Anti-phospho-Cleaved PARP	Rabbit polyclonal	1:1000*	Abcam (Cambridge, MA, USA)
Anti-phospho-ERK (Thr202/Tyr204)	Rabbit polyclonal	1:3000*	Cell Signaling (Temecula, CA, USA)
Anti-phospho-FAK (Tyr397)	Rabbit polyclonal	1:2000*	Cell Signaling (Temecula, CA, USA)
Anti-SNAP25	Mouse monoclonal	1:50	Santa Cruz (Santa Cruz, CA, USA)
Anti-Syntaxin 1A	Mouse monoclonal	1:50	Santa Cruz (Santa Cruz, CA, USA)
Anti-VAMP2	Rabbit polyclonal	1:200	Abcam (Cambridge, MA, USA)
Anti-β-actin	Mouse monoclonal	1:5000*	Sigma-Aldrich (St Louis, MO, USA)

* Dilution factor applied to western blot analysis. DSHB: Developmental Studies Hybridoma Bank.

Supplementary Table 3: Sequences of primers used in real-time PCR

Primer	Accession definition	Sequence 5′-3′ (sense/antisense)	Fragment size (bp)	Annealing temp (°C)
Glucagon	NM_008100.4	GAT CAT TCC CAG CTT CCC AG<br>CGG TTC CTC TTG GTG TTC AT	163	56.5
Ins1&2	NM_008386.3<br>NM_008387.4	GGC TTC TTC TAC ACA CCC A<br>CAG TAG TTC TCC AGC TGG TA	182	53.9
Itga3 (α3)	NM_013565.2	GCC ATC CGC CCT GCT ACT GT<br>AGT ACG GGC TGC AAG TTG TCC	219	60.9
Itga5 (α5)	NM_010577.3	GGC ACC CAA GGC TAA CAC TA<br>CGA ACT GTT GCT CCG AAC CA	204	57.7
Itga6 (α6)	NM_008397.3	TGG CTT CCT CGT TTG GCT ATG<br>GAA TCG GCT TCA CAT TAC TC	168	54.2
ItgaV (αV)	NM_008402.2	ACC TGG ACG TCG AAA GTC CC<br>CCG GCG GCT GGA TGA GCA TT	194	59.4
Itgb1 (β1)	NM_010578.2	GGC AAC AAT GAA GCT ATC GTG<br>TTC GGA TTG ACC ACA GTT GTC	257	55.1
Pdx1	NM_008814.3	CCA CCC CAG TTT ACA AGC TCG<br>GTA GGC AGT ACG GGT CCT CT	324	57.5
Snap25	NM_011428.3	GGC TTC ATC CGC AGG GTA AC<br>CTG GCG ATT CTG GGT GTC AAT	138	58.9
Syntaxin1A	NM_016801.3	AAG GAC AGC GAT GAC GAC GAC<br>TTC GGC AAT CTT GTC AAT AA	114	55.5
Syntaxin3	NM_001025307.1	GTC GGC ACA AGG ACA TCG TA<br>TGT TCT CAA TGC GGT CAA TCA	117	56.5
Vamp2	NM_009497.3	GAG CGG GAC CAG AAG TTG TC<br>TCC ACC AGT ATT TGC GCT TG	109	57.8
18S	NR_003278.3	GTA ACC CGT TGA ACC CCA TTC<br>CCA TCC AAT CGG TAG TAG CG	151	55.6
